# The Impact of Education and Culture on Poverty Reduction: Evidence from Panel Data of European Countries

**DOI:** 10.1007/s11205-023-03155-0

**Published:** 2023-06-14

**Authors:** A. Spada, M. Fiore, A. Galati

**Affiliations:** 1 Department of Economics, University of Foggia, 71121 Foggia, Italy; 2 Department of Agricultural, Food and Forest Sciences, University of Palermo, Viale Delle Scienze, 90128 Palermo, Italy

**Keywords:** Education, European countries, Poverty degree, Culture, Principal component analysis, Pooled OLS model, Fixed effect model, Random effect model

## Abstract

The 2030 Agenda has among its key objectives the poverty eradication through increasing the level of education. A good level of education and investment in culture of a country is in fact necessary to guarantee a sustainable economy, in which coexists satisfactory levels of quality of life and an equitable distribution of income. There is a lack of studies in particular on the relations between some significant dimensions, such as education, culture and poverty, considering time lags for the measurement of impacts. Therefore, this study aims to fill this gap by focusing on the relationship between education, culture and poverty based on a panel of data from 34 European countries, over a 5-year period, 2015–2019. For this purpose, after applying principal component analysis to avoid multicollinearity problems, the authors applied three different approaches: pooled-ordinary least squares model, fixed effect model and random effect model. Fixed-effects estimator was selected as the optimal and most appropriate model. The results highlight that increasing education and culture levels in these countries reduce poverty. This opens space to new research paths and policy strategies that can start from this connection to implement concrete actions aimed at widening and improving educational and cultural offer.

## Introduction

Poverty eradication has been the key objective for spans in many countries since that has been recognized as the greatest hostile issues ‘jeopardising balanced society socio-economic development’ (Balvociute, 2020). Poverty can be considered one of the core features of unsustainable socio-economic development and as a persistent phenomenon that can have upsetting effect on peoples’ lives (Bossert et al., 2022). For this reason, the extreme poverty removal, as well as the fight against inequalities and injustices, have been placed at the center, with climate change, of the 2030 Sustainable Development Goals. The nature of poverty is multidimensional and inequalities within and among countries is an obstinate origin for concern (Fund, 2015; Alvaredo et al., 2017; Alkire & Seth, 2015; Kwadzo, 2015). For its interpretation and measurement, the literature has added to the monetary approach of material deprivation, the social and subjective dimension of the human being (Bellani & D’Ambrosio, 2014; Maggino, 2015). As stated by Kwadzo (2015), it is possible to define three poverty measurements: monetary poverty, social exclusion, and capability poverty. Similarly, there are a lot of indicators measuring well-being and quality of life: Index of Happiness, Human Poverty Index and Human Development Index (Senasu et al., 2019; Spada et al., 2020; UNDP, 1990; Veenhoven, 2012; Watkins, 2007). All these indicators focus and start from education. For example, the Human Poverty Index (HPI) was introduced by the United Nations to complement the Human Development Index (HDI) and used, for the first time, in the 1997 Human Development Report. In 2010, it was replaced by the Multidimensional Poverty Index. The HPI focuses on the deprivation of three essential parameters of human life, already taken into account by the Human Development Index: life expectancy, education and standard of living (Alkire et al., 2015; UNDP, 1990).

Previous studies shown that education indicators have a large impact on a country’s poverty (Bakhtiari & Meisami, 2010; UNDP, 1990; Watkins, 2007) and that investing in health and education is a way to reduce income inequality and poverty. In addition, studies highlight that increasing equality and the quality of education is essential to combat economic and gender inequality within society (Walker et al., 2019). However, few studies provide empirical evidence on how education impacts on income inequality (Liu et al., 2021; Santos, 2011; Walker et al., 2019) and most of these studies analyses the poverty phenomenon neglecting the combined effect of various variables. Different dimensions of poverty have also empirically demonstrated a high degree of correlation (Kwadzo, 2015). In addition, the literature review analysis highlighted a gap in quantitative studies, especially on the paths between some relevant dimensions, such as education, culture and poverty, considering time lags for the measurement of impacts. In light of this, the main objectives of this study are: (i) To identify over the five-year period considered (2015–2019), with what delay and with what magnitude and sign, the poverty is influenced by some indicators representative of the educational and cultural dimension; and (ii) Consequently, better calibrate education policies in European countries, in order to achieve a reduction in the poverty rate in the short term, in compliance with the objectives of the 2030 Agenda.

The rest of the paper is organized as follows. A literature review regarding the relation between poverty, education and inequalities is presented in Sect. 2. The Sect. 3 enlightens research gaps linked to the aims of this study and hypothesis to corroborate. Section 4 defines data and summarizes the methodological approach used to reach the work’s aims. Results are presented and discussed in Sect. 5. Finally, the last section sets out our main conclusions by highlighting limitations of the study and future directions.

## Theoretical Framework

### The Core Role of Education

Over the last decades it is possible to individuate in the EU-28 a quickly growing portion of the population having income below 60% of the median disposable income. In addition, there is a share of the population has been becoming more impoverished (Balvociute, 2020; EUROSTAT Statistic Explained, 2019). In same way, it is possible to speak about “poverty trap”, a mechanisms whereby countries are poor and persist poor: existing poverty appears a straight cause of poverty in the future (Knight et al., 2009; Kraay & McKenzie, 2014). Aspects such as accommodation, education, medical and material services are considered essential. In particular, an increasing number of empirical studies have supported the positive effects of education on the creation of wealth by individuals and on promoting economic effective and fair development (UNESCO & Global Education Monitoring Report, 2017; Walker et al., 2019; Xu, 2016; Zhang, 2020). A research note by European Commission (2015) shows that individuals with primary education remain the most vulnerable in all EU countries (with a risk of poverty ranging from 13%—Netherlands—to 56% Romania). Even the Millennium Development Goals (MDGs), the Poverty Reduction Strategy Papers (PRSP) endorsed by the World Bank and ‘Education for All’ program (UNESCO, 2007) emphases the significant role of education (Awan et al., 2011). A diverse balance can be possible and policy efforts to interrupt the poverty trap might have long-term effects. In this framework, the model proposed by Santos (2011) shows that a policy oriented towards aligning the quality of education would reduce initial inequalities. In light of this, Shi & Qamruzzaman, (2022) in a recent work, study, by means of numerous econometrical methods, the tie between investments in education, financial inclusion, and poverty decrease for the period 1995–2018 in 68 nations, underlining the role of education-backed poverty mitigation public policies that need to be more targeted. Several studies demonstrate that level of poverty and education are strictly related. For instance, Bossert et al. (2022) by focusing on Atkinson-Kolm-Sen index, that measures the percentage income gap of the poor that can be attributed to inequality among the poor (Sen, 1973, 1976), emphasized the close relation between poverty and inequality. Consistent with previous studies, Lenzi and Perruca (2022) demonstrate that tertiary educated people report higher ranks of life satisfaction. This link is even more marked in rural territories where education is recognised as an important tool for reducing poverty as it allows the acquisition of skills and productive knowledges which increase people’s productivity and their earnings (Tilak, 2002). A recent report of the United Nations (2021) underlines how the reduced access to educational and health services in rural areas becomes a barrier, determining the difficulty of people living in these areas to found employment in well-paid professions contributing to economic growth (Chmelewska and Zegar, 2018). However, as Liu and colleagues (2021) find, different levels of education have distinct effects on poverty in rural areas of China and that the latter is driven not only by factors within the region but also by the level of poverty in the surrounding regions. In addition, numerous empirical evidences reveal a link between educational level and income inequalities in several geopolitical contexts. Bakhtiari and Meisami (2010), in a work of over 10 years ago, makes use of a panel data set of 37 Islamic countries (eight time periods) to study income inequality along with a model of poverty, with the main variables as income level, health status, education and savings. Findings show that enhancing the health and education can reduce income inequality and poverty. Likewise, as Arafat and Khan (2022) underline the high level of education not only contributes to reducing the degree of poverty but improves the conditions of mental, social and emotional well-being compared to poorly educated families. After about 10 years, similar works by Wani and Dhami (2021) and Sabir and Aziz (2018) reach the same results investigating the SAARC (South Asian Association for Regional Cooperation) countries and 31 developing countries (by employing the System Generalized Method of Moments). In several cases, and especially in rural areas, poverty is linked to the lower level of household income compared to urban areas, resulting in differences in access to basic goods and services to meet personal needs (Chmelewska and Zegar, 2018). In this territories household income level is directly associated with food security, in fact, an increase in the level of income reduces food insecurity (Chegini et al., 2021). However, as evidenced by other authors (Kirkpatrick et al., 2020; Kusio & Fiore, 2022), access to education can help to overcome the migration of young people and geographical isolation and inaccessibility that characterize the poor areas (Kvedaraite et al., 2011). In turn, young, educated people affect entrepreneurial attitudes. Walker et al. (2019) in the recent report ‘*The Power of Education to Fight Inequality. How increasing educational equality and quality is crucial to fighting economic and gender inequality*’ show how education can be emancipating for individuals, and it can play the role of a ‘leveler and equalizer within society’. Education interrupts obstinate and rising inequality by promoting the development of more decent work, rising incomes for the poorest people: it can aid to endorse long-lasting, wide-ranging economic growth and social cohesion.

Gradstein and Justman (2002) underlined the role of education in shaping the social cohesion that can assure equality between individuals. Universal free education enhances people’s earning power, and can bring them out of poverty. Low levels of education hamper economic growth, which in turn slows down poverty reduction (UNESCO, 2017; Global Education Monitoring Report, 2019) estimates that each year of schooling raises earnings by around 10%;53 this figure is even higher for women. In Tanzania, having a secondary education reduces the chances of being poor as a working adult by almost 60%. According to a study by UNESCO and the Global Education Monitoring Report (2019), if all adults finished secondary school, 420 million individuals would be lifted out of poverty. The convergence of crises deriving first from COVID-19 then from climate change, and conflicts, are generating extra impacts above all on poverty, nutrition, health and education affecting all the Sustainable Development Goals (SDGs).

Equilience, a synchratic neologism composed of Equity + Resilience, that is resilient systems in respect of equity as a balancing of the different interests of the parties. Recent research (Berbés-Blázquez et al., 2021; Williams et al., 2020; Contò and Fiore, 2020) highlight the crucial importance to promote the ‘marriage’ between equity and resilience.

### Aims of Study and Hypothesis

This research is potentially the first study to investigate the relationship between educational, cultural factors and poverty in European countries.

The main research directions are as follows: (i) To assess the impact of education and culture (expressed by the following indicators: *Cultural employment, Total educational expenditure, Graduates in tertiary education, Number of enterprises in the cultural sectors, Tertiary educational attainment*) upon poverty (indicated by *Persons at risk of poverty or social*); (ii) To compare the strength and direction of the relationships between the variables considered in two temporal situations, i.e. with zero lag, and with lag equal to one year. The data cover the period 2015–2019 and were extracted from the Eurostat database.

In the light of the above discussion, of the literature review analysis, and of the theoretical frameworks examined this study explores the following research hypotheses with regard to the European context:

#### H_1_

Education and culture have an inverse impact on the levels of poverty.

Our second hypothesis states:

#### H_2_

The association between cultural, educational variables and poverty, in the short term is more intense if we consider a delay of one-year.

### Data

The dataset is a balanced panel of annual observations for 34 European countries and covers the period from 2015 to 2019. On the basis of literature findings, our analysis focused on the following dimensions: education, income inequality and poverty.

Thereby, the variables considered for our investigation are as follows:

- Poverty indicator: *Persons at risk of poverty or social exclusion* (% of population, thousand persons; hereinafter labelled with PRP);
- Education and cultural indicators: *Cultural employment* (thousand persons); *Total educational expenditure* (million euros); *Graduates in tertiary education* (‰ of population;); *Number of enterprises in the cultural sectors*(number) *Tertiary educational attainment* (‰ of population). Respectively, hereinafter they will be labelled with CE, TEE, GTE, NEC and TEA.

The indicators have been extracted from the Eurostat database. The summary statistics are reported in Table 1. In the selected time period, Iceland is the country that shows the lowest values with respect PRP (12.08%). Instead, the country showing the worst performance is Romania (PRP = 41.60%). With regard to the education indicators, Germany holds the highest values for both CE (81,661.48 thousand persons) and TEE (30.588.86 million euros), highlighting great attention to education issues. Instead, in Eastern Europe (Montenegro, Romania, and Hungary) the indicators pertaining to the education area take on more penalized values. Italy is the country that boasts the largest number of enterprises in the cultural sector (NEC = 179,136.8), thanks also to the artistic beauties of which this country is rich. As far as the tertiary education level is concerned, the highest value of is held by Cyprus while the lowest by Romania (respectively TEA = 57.34 and TEA = 25.26). For subsequent processing, since the variables considered are both in the form of ratios and counts, all data were converted to natural logarithms.

**Table 1:** Summary statistics for the Eurostat datasets, 2015–2019

Country	PRP (% of people)	CE (thousand persons)	TEE (million euros)	GTE (‰ of people)	NEC (number)	TEA(‰ population)
	Mean ± SD	Mean ± SD	Mean ± SD	Mean ± SD	Mean ± SD	Mean ± SD
Austria	16.90 ± 0.27	174.32 ± 7.00	2,321.94 ± 349.82	22.44 ± 0.64	17,206,20 ± 756,05	40,14 ± 1,10
Belgium	21.26 ± 0.96	192.92 ± 12.66		13.88 ± 0.42	40,883,40 ± 1793,72	45,56 ± 1,88
Bulgaria	37.70 ± 4.60	85.64 ± 1.68	406.27 ± 34.87	14.08 ± 0.38	10,622,60 ± 298,85	32,94 ± 0,82
Croatia	22.90 ± 1.44	54.90 ± 4.40	366.78 ± 50.34	18.16 ± 1.22	7277,80 ± 1290,32	33,44 ± 2,00
Cyprus	20.90 ± 1.95	13.18 ± 0.81	103.86 ± 4.97	10.10 ± 0.10	2419,60 ± 246,29	57,34 ± 2,15
Czechia	12.28 ± 0.45	198.36 ± 9.17	1,019.77 ± 161.00	16.72 ± 0.43	49,046,75 ± 4227,15	32,66 ± 1,06
Denmark	17.74 ± 0.51	123.68 ± 2.37	3,888.45 ± 130.80	22.62 ± 1.41	13,342,40 ± 600,75	45,2 ± 1,52
Estonia	23.46 ± 0.25	35.00 ± 1.50	242.30 ± 0	16.08 ± 0.46	3538,40 ± 329,77	40,04 ± 1,17
Finland	16.28 ± 0.59	122.56 ± 6.58	2,703.90 ± 139.27	23.80 ± 0.90	10,190,00 ± 99,94	40,7 ± 0,75
France	18.34 ± 0.48	917.60 ± 45.72	16,419.90 ± 0	26.08 ± 1.05	162,416,80 ± 7866,12	45,74 ± 1,76
Germany	18.88 ± 1.09	1,661.48 ± 14.06	30,588.86 ± 3,489.47	21.10 ± 1.85	132,197,40 ± 3848,77	31,4 ± 1,46
Greece	31.30 ± 1.58	122.84 ± 8.41		17.38 ± 0.36	30,813,80 ± 1819,10	41,76 ± 1,16
Hungary	25.14 ± 4.73	154.76 ± 6.55	1,079.95 ± 151.82	12.28 ± 0.19	29,958,80 ± 3268,67	30,78 ± 0,76
Iceland	12.08 ± 0.64	11.46 ± 0.49	393.85 ± 0.21	16.60 ± 1.12	2483,75 ± 137,24	45,1 ± 3,22
Ireland	22.60 ± 2.03	77.22 ± 1.69	479.40 ± 190.51	33.04 ± 3.01	13,769,33 ± 257,36	54,92 ± 0,89
Italy	26.48 ± 1.57	808.94 ± 29.78	9,435.56 ± 753.67	14.74 ± 1.21	179,136,80 ± 3136,58	26,62 ± 1,17
Latvia	28.36 ± 1.17	35.82 ± 3.52	236.65 ± 15.64	13.22 ± 0.63	5022,80 ± 248,33	41,8 ± 1,39
Lithuania	28.64 ± 1.85	52.24 ± 2.78	397.98 ± 81.01	19.02 ± 0.82	11,739,60 ± 1246,77	55,22 ± 0,38
Luxembourg	19.40 ± 0.72	13.62 ± 1.12	302.20 ± 30.66	3.80 ± 0.23	1593,00 ± 29,94	52,58 ± 2,33
Malta	20.36 ± 1.21	10.82 ± 1.95	50.16 ± 5.91	13.18 ± 1.66	1696,67 ± 143,67	36,36 ± 3,82
Montenegro	41.38 ± 2.84	8.08 ± 0.90				35,04 ± 2,71
Netherlands	16.46 ± 0.11	397.92 ± 21.41	2,862.66 ± 195.29	12.80 ± 0.80	93,253,80 ± 10,543,68	46,72 ± 1,69
North Macedonia	37.36 ± 2.85	23.30 ± 1.56		7.68 ± 0.50	2150,40 ± 114,32	33,04 ± 1,91
Norway	15.18 ± 0.60	102.70 ± 1.31	5,908.90 ± 154.21	15.68 ± 1.17	18,001,20 ± 707,56	48,68 ± 0,40
Poland	19.58 ± 1.94	555.72 ± 21.88	3,961.14 ± 626.49	21.50 ± 1.30	80,647,80 ± 8488,26	43,46 ± 0,15
Portugal	23.48 ± 2.22	152.92 ± 10.54	1,137.20 ± 0	19.96 ± 1.00	33,220,00 ± 2113,49	34,92 ± 1,61
Romania	41.60 ± 4.03	135.52 ± 6.19	697.46 ± 150.94	15.56 ± 1.21	18,191,00 ± 2282,65	25,26 ± 0,38
Slovakia	16.06 ± 1.09	68.22 ± 6.15	520.13 ± 53.81	14.78 ± 1.47	13,580,60 ± 1419,18	35,24 ± 3,10
Slovenia	16.06 ± 1.56	44.84 ± 2.94	482.67 ± 19.66	22.82 ± 5.89	9332,80 ± 521,58	42,62 ± 1,79
Spain	27.70 ± 1.08	672.10 ± 38.32	9,303.50 ± 161.39	21.66 ± 0.55	127,827,00 ± 3452,69	43,08 ± 2,35
Sweden	17.84 ± 0.47	240.04 ± 7.50	8,791.07 ± 175.78	15.30 ± 0.37	52,449,80 ± 587,60	47,44 ± 0,68
Switzerland	18.10 ± 0.54	248.18 ± 2.89		20.84 ± 0.79		49,86 ± 2,36
Turkey	30.24 ± 3.30	637.76 ± 31.38	2,244.40 ± 647.71	12.48 ± 0.43		30,52 ± 2,86
United Kingdom	22.40 ± 0.65	1,476.72 ± 24.67	13,143.70 ± 0	24.04 ± 1.64	101,032,25 ± 2313,12	47,74 ± 1,04

## Methodology

The methodological approach used is based on linear panel data models including the simple Pooled Ordinary Least Square (pooled OLS) model, the Fixed Effects (FE) model and the Random Effects (RE) model. Before proceeding with the application of the linear models, the correlation matrix between the variables taken into consideration was performed and subsequently, to avoid multicollinearity problems and distorted estimates, the study, based on the principal component analysis (PCA), used two indicators related to education and culture. According to Jolliffe and Cadima (2016), through PCA starting from a set of correlated variables, a set of uncorrelated variables is obtained, known as Principal Components (PC). In PCA, only common factors that have an eigenvalue greater than one or greater than the mean should be kept (Jolliffe, 2002; Kaiser, 1974). In this study PCA allowed to obtain the following indicators: EDU1, which includes CE, NEC, TEE, and EDU2, composed of TEA and GTE. These indicators have been incorporated into the panel data models, replacing the original variables.

The first linear panel data model adopted is the pooled OLS, which assumes no heterogeneity between countries, whose equation is as follows:

1 $${\text{ln}}PRP_{it} = \alpha + \beta EDU_{it} + \varepsilon_{it} {\text{ i}}:1,2,...,{\text{N t}}:{ }1,2,...,{\text{ T }}$$

where *ln*PRP is the natural logarithm of the poverty indicator, α is the intercept, EDU is composed of the principal components extracted, ε is the error term, i denotes statistical units, in this case countries, and *t* denotes the time index.

The second model adopted is FE which controls for cross-country heterogeneity and is expressed as:

2 $${\text{ln}}PRP_{it} = \alpha_{i} + \beta EDU_{it} + \varepsilon_{it} \quad{\text{ i}}:1,2,...,{\text{N t}}:{ }1,2,...,{\text{ T}}$$

where $\alpha_{i}$ is the regional specific parameter denoting the fixed effect. The basic intuition of the FE model is that $\alpha_{i}$ does not change over time.

Finally, the third model is RE denoted as;

3 $${\text{ln}}PRP_{it} = \alpha + \beta EDU_{it} + \omega_{it}\quad {\text{ i}}:1,2,...,{\text{N t}}:{ }1,2,...,{\text{ T}}$$

In the RE model, variations between units are assumed to be random and uncorrelated with the independent variables in the model.

To verify the two research hypotheses, for each of the three models (pooled OLS, FE and RE) two versions were calculated, with lag 0 and lag 1 year. In the model at lag 0 the variables are synchronous, while in the model at lag 1 principal components enter the equation with a one-year lag compared to PRP. The choice of the reference model between pooled OLS, FE and RE is based on several tests. In choosing between FE and pooled OLS, the study applies the F-test. A p-value of less than 5% indicates that there are important country effects that OLS fails to detect, and that thus neglecting unobserved heterogeneity in the model can lead to estimation errors and inconsistencies. The study also tests which is better between the OLS and RE model using the Breusch-Pagan (BP)-Langragian Multiplier (LM) test. The null hypothesis of the BP-LM test is that there is no substantial variance between regions. A probability value of less than 5% for the BP-LM test indicates that the RE model is appropriate and the OLS pool is not. Finally, the Hausman test χ^2^ is also performed to compare the FE model and the RE model. According to Algieri and Mannarino (2013), the Hausman test χ^2^ aims to identify a violation of the RE modelling hypothesis. In this test, the alternative hypothesis is that the FE model is preferable to the RE model, while the null hypothesis is that both models produce similar coefficients. A p-value greater than 5% denotes that both FE and RE are reliable, but the RE model is more efficient because it uses a lower degree of freedom. We also test for heteroskedasticity in the FE model using the modified Wald test developed by Lasker and King (1997). The null hypothesis of this test is that the variance of the error is similar for all countries (Amaz et al., 2012). All statistical analyses were conducted in Stata 17.0 (Stata Corp LP, College Station, Texas, USA). A critical value of *p* < 0.05 was specified a priori as the threshold of statistical significance for all analyses.

## Results

The relationships between the variables, measured by Pearson’s linear correlation coefficient, is shown in Table 2. It is noted that the PRP variable is negatively correlated with all the other panel variables, albeit with modest correlations. Instead, TEE shows a high positive correlation with NEC (*r* = 0.963 and *r* = 0.903, respectively). There is also a high correlation between NEC and TEE (*r* = 0.857). Therefore, in the light of the results, to exclude the problem of multicollinearity between the covariates, we proceeded to analyse the principal components.

**Table 2:** Pearson correlation coefficient

	PRP	CE	TEE	GTE	TEA	NEC
PRP	1.000					
CE	− 0.116	1.000				
TEE	− 0.169	0.903*	1.000			
GTE	− 0.209*	0.474***	0.388***	1.000		
TEA	− 0.384***	− 0.156*	− 0.198*	0.087	1.000	
NEC	− 0.082	0.963***	0.857***	0.500*	− 0.166*	1.000

*p < 0.05, **p < 0.01, ***p < 0.001

Table 3 shows the results of principal component analysis. On the basis of these results, the need to maintain the first two principal components is highlighted, since their eigenvalues are greater or very close to 1 and cumulatively represent the 84% of the information. They will be labelled as EDU1 and EDU2 respectively. EDU1 refers to TEE, CE and NEC, i.e. it refers to a cultural dimension of the country and therefore, even if not strictly connected to the school environment, with an important educational role, while the EDU2 component referring to GTE and TEA, is more closely related to the school.

**Table 3:** Principal component analysis: factor loading, eigen value and variance explained

Variables	Comp1	Comp2	Comp3	Comp4	Comp5
TEE	0.5041	0.1435	0.3466	− 0.7374	− 0.2478
GTE	0.3329	0.4747	− 0.8123	− 0.0476	− 0.0423
CE	0.5433	− 0.0216	0.1581	0.1705	0.8064
NEC	0.5292	− 0.0120	0.2005	0.6316	− 0.5298
TEA	− 0.2446	0.8680	0.3936	0.1609	0.0768
Eigenvalue	3.27227	0.929618	0.647951	0.129206	0.0209512
Proportion	0.6545	0.1859	0.1296	0.0258	0.0042
Cumulative	0.6545	0.8404	0.9700	0.9958	1.0000

Table 4 shows the results of the three econometric models (pooled OLS, FE, RE) on the link between education, culture and poverty. It is observed that all models converge in showing that poverty decreases with increasing education and culture. In particular, the EDU1 indicator always shows a negative coefficient, and this relationship is statistically significant in the model fixed at lag 0 and lag 1 (respectively *b* = − 0.3804, *p* < 0.001; *b* = − 0.3925, *p* < 0.001). Furthermore, for EDU1, in all three econometric models it can be noted that the coefficients are higher in absolute value passing from lag 0 to lag 1, highlighting that the impact between cultural and educational tools and poverty reduction occurs with a delay, perhaps necessary to have positive results. Also, the EDU2 indicator always shows a negative coefficient and this relationship is statistically significant in all three models, both at lag 0 and at lag 1 (for all *p* < 0.001). To discern the econometric model that best fits the data, as a first step the F-test allows you to choose between the OLS and FE models. The value F = 80.09 for lag 0 and F = 109.61for lag = 1, (for all *p*-value < 0.001), indicates in both cases that the FE model is more suitable than the pooled OLS. This demonstrates that in the relationships examined time plays an important role, which a simple OLS model may fail to capture, i.e. EDU1 and EDU2 have an effect on poverty decrease that changes over time. The choice between the RE model and the pooled OLS was instead based on the BP LM test, which suggests that the RE model is more suitable than the pooled OLS. Finally, the Hausman test χ^2^ allows to identify which between FE and RE is more suitable: The value χ^2^ = 15.95 at lag 0 and χ^2^ = 13.40 at lag = 1, (for all *p*-value < 0.001) suggests that the FE model is more suitable than the RE model, indicating the presence of non-random differences between countries or over time. The model that best fits the examined panel of data is therefore the FE model.

**Table 4:** Pooled OLS, Fixed Model, Random Model, at lag 0 and at lag 1

Variable	Model 1 (lag 0 year)			Model 2 (lag 1 year)		
	Pooled OLS	Fixed effect	Random Effect	Pooled OLS	Fixed effect	Random Effect
*PRP*						
EDU1	− 0.004	− 0.380***	− 0.026	− 0.013	− 0.393***	− 0.032
EDU2	− 0.112***	− 0.141**	− 0.178***	− 0.105***	− 0.163**	− 0.168 ***
Constant	3.039***	3.039***	3.035***	3.026***	3.013***	3.041***
F-stat		20.98***			18.01***	
F test		80.09***			109.61***	
Wald	22.82		22.82***	20.14***		20.14***
Hausman test χ^2^		15.95***			13.40***	
BP-LM			129.21***			107.04***

**p < 0.01, ***p < 0.001

In light of these results, as supposed in hypothesis *H*_*1*_, it is evident that education and culture play a significant role in poverty reduction. Furthermore, as supposed by hypothesis *H*_*2*_ and based on the FE model which was found to be the most suitable, this impact is more intense if one considers a year of delay, above all for cultural and educational variables relating to a dimension that is not strictly scholastic.

## Discussions and Conclusions

The present study analysed the relationship between education, culture and poverty for 34 countries, over the period 2015–2019. The findings indicate that rising education and culture levels in these nations reduce poverty. The model also highlighted that this relationship is weaker if we consider a contemporaneity of the values of the variables (at lag 0), while it is strengthened if we consider a time interval of one year.

As policy-makers regularly disclose the consequences of unfair development by identifying problems requiring solutions built on evidence-based guidelines, these results can have interesting and fruitful implications. By concluding, education appears, in line with other studies (Sabir & Aziz, 2018; Xu, 2016), one of the best effective methods to eradicate poverty. In line with the work by Walker et al., (2019), investing in universal-free-public education for all the persons can close different circles: the gap between rich and poor people, between women and men, between poor and rich areas within a country and among countries. In addition, education appears crucial to fight inequalities across the world. The results appear also consistent with the UN report (2021) that emphasizes the importance of the access to educational and health services in marginal poor areas to improve and contribute equal economic growth and reduce poverty (Chmelewska and Zegar, 2018; Bakhtiari & Meisami, 2010; Wani & Dhami, 2021). The same findings come from the work by Peng (2019) based on data from poor Chinese provinces showing that education has steady and positive impacts on farmers’ income, and the outcome of growing income in poor zones is higher than in other areas.

All in all, as evidenced by the European Commission (2015), the means to diminish the risk of poverty appears ‘straight-forward: go to school, get a job’. Clearly, these implications have to consider conditions and country environment. In line with previous research (Noper Ardi & Isnayanti, 2020; Walker et al., 2019), these results highlight that education can have an immediate impact on income inequalities and poverty; on the other hand, education (and public spending on it) has a longer-term impact on inequality through its effects in enhancing future salaries and chances. Indeed, as stated by some notable researchers (Kraay & McKenzie, 2014), the ‘more-likely poverty traps’ need action in less-traditional policy areas. The scholars have to further perfect the theoretical concepts and policy standards of poverty alleviation through education (Shi & Qamruzzaman, 2022).

This paper reinforces the conclusions deriving from other research (Mou and Xu, 2020; Assari et al., 2018; Batool and Batool, 2018) that are to give evidence of how education can forecast coming ‘Emotional Well-Being’ thus decreasing the inequalities by means of more generous policies and strategies. The latter can support international experience-based education (Xu, 2016).

In the following research phases, other variables can be inserted to improve the specifications of the model and also verify the existence of homogeneous groups of countries. In addition, a distinction between urban and rural areas to highlight the link between income, education and poverty and differences could enrich the literature and provide useful information to guide national policies in a targeted way. Regarding possible limitations of the paper, it is possible to notice a time period limited for missing data and health variables are missing.

The ‘dark’ side of this conclusions is considering the effects of the COVID19 pandemic that has increased on one hand the online teaching and training: on the other hand, education has become more difficult in remote, rural and/or marginal areas due to connections and hardware limitations.

Therefore, nowadays strategies, models and polices focusing on equi-lience (equity and resilience) processes can promote the creation of a different balance between the needs of sustainable growth and those of social, fair and environmental development (Fiore, 2022). Therefore, developing a strategy to convey a trained, skilled and well-supported workforce, investing in relevant and fair teaching resources, ensuring funds and building better liability mechanisms from national to local levels can be significant and fair paths to reduce poverty and inequalities. These strategies have to be aimed at developing national education plans that try to identify pre-education existing inequalities in order to arrange actions in poorer rural and marginalized districts or regions.
